# Comparison of respiratory muscle strength through manovacuometry in the early postoperative period of bariatric surgery by laparotomy and laparoscopy

**DOI:** 10.1590/0100-6991e-20223056-en

**Published:** 2022-07-08

**Authors:** ARIELI LUZ RODRIGUES BARETTA, ALEXANDRE COUTINHO TEIXEIRA DE FREITAS, CAROLINA MOCELLIN, MARIA PAULA CARLINI CAMBI, ANDRÉ RICHTER RIBEIRO, CLÁUDIA GISSI DA ROCHA FERREIRA, GIORGIO ALFREDO PEDROSO BARETTA

**Affiliations:** 1 - Universidade Federal do Paraná, Disciplina de Clínica Cirúrgica - Curitiba - PR - Brasil; 2 - Clínica de Cirurgia Bariátrica Dr. Giorgio Baretta, Departamento de Fisioterapia - Curitiba - PR - Brasil; 3 - Clínica de Cirurgia Bariátrica Dr. Giorgio Baretta, Departamento de Cirurgia - Curitiba - PR - Brasil; 4 - Hospital São Lucas, Departamento de Cirurgia Bariátrica - Campo Largo - PR - Brasil

**Keywords:** Bariatric Surgery, Maximal Respiratory Pressures, Laparotomy, Laparoscopy, Cirurgia Bariátrica, Pressões Respiratórias Máximas, Laparotomia, Laparoscopia

## Abstract

**Introduction:**

surgical treatment of obesity causes important changes in respiratory mechanics.

**Aim:**

Comparatively analyze respiratory muscle strength in post bariatric patients underwent to gastric bypass by laparotomy and laparoscopy during hospital stay.

**Methods:**

observational study with a non-randomized longitudinal design, of a quantitative character. Data were collected from 60 patients with BMI 40Kg/m^2^, divided in laparotomy group (n=30) and laparoscopy group (n=30). Smokers, patients with previous lung diseases and those unable to perform the exam correctly were excluded. Both groups were evaluated at immediate postoperative, first and second postoperative days with manovacuometry for respiratory muscle strength and visual analogue pain scale.

**Results:**

the sample was homogeneous in age, sex and BMI. Reduction in maximal respiratory pressures was observed after surgery for those operated on by laparotomy, no return to baseline values on discharge day on the second postoperative day. This group had also more severe pain and longer operative time. There was no difference in respiratory pressure measurements after surgery in the laparoscopy group.

**Conclusion:**

conventional bariatric surgery reduces muscle strength in the postoperative period and leads to more intense pain during hospitalization when compared to the laparoscopy group.

## RESUMO

**Introdução:**

o tratamento cirúrgico da obesidade acarreta importantes alterações na mecânica respiratória.

**Objetivo:**

analisar comparativamente a força muscular respiratória em pacientes submetidos à cirurgia bariátrica do tipo bypass gástrico por laparotomia e por videolaparoscopia durante o internamento cirúrgico.

**Métodos:**

estudo observacional com delineamento longitudinal não-randomizado, de caráter quantitativo. Foram coletados dados de 60 pacientes com índice de massa corporal igual ou superior a 40Kg/m^2^, candidatos a cirurgia bariátrica e divididos em grupo 1, para os operados por laparotomia (n=30), e grupo 2, para os operados por videolaparoscopia (n=30). Foram excluídos os tabagistas, os pacientes incapazes de executar o exame de forma correta e os portadores de doenças pulmonares prévias. Ambos os grupos foram avaliados no pré-operatório imediato, no primeiro e no segundo dias de pós-operatório através do teste de manovacuometria para a força muscular respiratória e da escala visual analógica de dor.

**Resultados:**

a amostra foi homogênea em relação à idade, índice de massa corporal e sexo. Foi observado redução das pressões respiratórias máximas após a cirurgia para os operados por laparotomia, sem retorno aos valores basais no dia da alta hospitalar no segundo dia pósoperatório. Esse grupo também cursou com dor mais intensa e maior tempo cirúrgico. Não houve diferença das medidas de pressão respiratória após a cirurgia no grupo operado por laparoscopia.

**Conclusões:**

a cirurgia bariátrica pela via convencional reduz a força muscular respiratória no pós-operatório e cursa com dor mais intensa durante a internação cirúrgica em relação à via laparoscópica.

## INTRODUCTION

Obesity is a chronic disease of multifactorial etiology that causes several damages to individuals’ health^1^ ^,^ ^2^. It is considered by the WHO as a worldwide epidemic and it is estimated that by 2025 one billion adults will be affected by it^3^.

Surgical treatment of obesity has been documented as the most effective in the long term and the one with the best results in improving quality of life and remission of comorbidities^4^ ^,^ ^5^. However, despite being an efficient and relatively safe approach, bariatric surgery by the conventional route has significant complications rates, ranging from 3.6% to 30% for complications of pulmonary origin^6^ ^-^ ^8^. Procedures in the upper abdomen cause important changes in respiratory mechanics due to the use of anesthetics, neuromuscular blockers and analgesics, surgical trauma, loss of integrity of the abdominal muscles, and postoperative pain^8^ ^,^ ^9^. On the other hand, laparoscopic or minimally invasive surgery has revolutionized the surgical treatment of obesity due to lesser operative trauma. However, respiratory function can also be compromised, especially due to the presence of pneumoperitoneum. In addition, the beneficial effects of laparoscopic surgery may be less pronounced in the presence of obesity, with an incidence of pulmonary complications around 1.6%^7^ ^,^ ^10^.

The behavior of respiratory muscle strength in these patients is still poorly understood. It is important to assess the inspiratory and expiratory muscle strength in the preoperative and postoperative periods of bariatric surgery, since respiratory muscle dysfunction is one of the causes of pulmonary complications^11^ ^,^ ^12^. The objective of this study is to evaluate and to compare respiratory muscle strength in morbidly obese individuals undergoing bariatric surgery by the conventional and by the laparoscopic routes during hospitalization for obesity surgical treatment.

## METHODS

This research was submitted to, evaluated, and approved by the Ethics in Research Committee of the Health Sciences Sector of the Federal University of Paraná under number CAAE:69704217.0.0000.0102. All research participants signed an Informed Consent Form.

For the present study, we selected and collected data from 30 patients who were candidates for bariatric surgery by laparotomy, from the bariatric surgery outpatient clinic of Hospital São Lucas de Campo Largo, and from 30 candidates for laparoscopic bariatric surgery from the private clinic of one of the researchers. We divided subjects into two groups: group 1, bariatric surgery by laparotomy; and group 2, laparoscopic bariatric surgery.

Inclusion criteria were age between 18 and 65 years, both sexes, ability to understand the study procedures, voluntarily agreeing to participate in the study and signing the informed consent, being morbidly obese (BMI ≥40kg/m^2^), and undergoing a gastric bypass-type gastroplasty.

Exclusion criteria were non-availability to the research protocol, inability to understand the study procedures, obesity grade II (BMI <40kg/m^2^), extreme age (under 18 years and over 65 years), smokers, previous pulmonary disease, and unwillingness to sign the informed consent form.

A single researcher evaluated all participants, having performed all procedures. We retrospectively collected data on history of lung disease, sex, age, height, weight, body mass index, and surgical time. We also compared the two groups in terms of respiratory pressures and pain scale.

The manovacuometry assessment was based on the guidelines of the American Society/European Respiratory Society (ATS/ERS) and the Brazilian Society of Pulmonology and Tisiology (SBPT)^13^ ^,^ ^14^. To assess maximal inspiratory pressure (MIP) and maximal expiratory pressure (MEP), we used an analog manovacuometer (M120 - GLOBALMED), with a variation capacity from -300 to +300 cmH2O, with scale intervals of 4 cmH2O. The patient was positioned seated at 90 degrees, feet on the floor, body relaxed, nose occluded with a nose clip to prevent air leakage during the procedure. To measure the MIP, we instructed participants to perform a maximum expiration reaching the residual volume, then connected the mouthpiece between the lips followed by a deep inspiratory effort, until the measurement pointer stabilized, held for two seconds. To measure MEP, participants performed a maximal inspiration until reaching total lung capacity, where the mouthpiece was connected between the lips, followed by a deep expiratory effort, sustained for two seconds. Individuals carried out five repetitions of maximal inspiration and expiration. To be included in the study, they must have been considered technically acceptable and reproducible and with values close to each other (≤10%). We considered the highest measures of MIP and MEP in the analysis. The first manovacuometry evaluation happened on the day before the surgical procedure. The same measurements took place on the first and second postoperative days, the latter being the date of hospital discharge. Also in the postoperative period, patients were evaluated daily using the VAS visual analogue pain scale^15^. If the reported pain intensity was greater than 5, analgesics were administered as prescribed by the physician. The evaluation through manovacuometry was only started after 30 minutes and if the patient reported pain below five on the visual scale.

Both groups underwent conventional respiratory physiotherapy on the first and second postoperative days, with exercises for lung re-expansion through ventilatory patterns, respiratory incentive, circulatory prophylaxis, and ambulation.

We tabulated the collected data in an Excel-Microsoft Office 2007 spreadsheet and analyzed it using the SPSS version 22.0 software. The variable sex was evaluated using the test for comparing two proportions. We evaluated the other data with the Student’s t or Mann-Whitney tests, according to the results of the preliminary analysis of the Kolmogorov-Smirnov normality test and Levene’s test for homogeneity of variance. The significance level adopted was α<0.05.

## RESULTS

Of the 60 patients selected, we excluded three from group 1 due to cancellation of the surgery and two from group 2 for refusing to perform respiratory pressure measurement in the second postoperative day. Thus, the total sample reached 55 patients, 27 patients in group 1 and 28 in group 2.

Most patients included were female (Table 1). There were 24 women in group 1 and 25 in group 2 (p>0.05). In group 1, the mean age was 37 years, the mean BMI was 44Kg/m^2^, and the mean surgery time was 118 minutes. In group 2, the mean age was 32 years and the mean BMI, 43Kg/m^2^. The mean surgery time was 57 minutes, shorter than that of group 1 (p<0.0001), as shown in Table 1.

**Table 1:** Demographic data and surgical time in minutes.

	Group 1 (n=27)	Group 2 (n=28)	p
Sex			
Female	24	25	0.96
Male	3	3	0.96
Age	37±9	32±9	0.06
BMI	44,0±4,1	43,1±3,6	0.38
STmin	118±16	57±9	<0.0001

n: number of subjects; p: p-value; BMI: body mass index; STmin: surgical time in minutes.

Maximum inspiratory pressures were lower in group 1 compared with group 2, both in the preoperative assessments and in the assessments on the first and second postoperative days (Table 2). Maximum expiratory pressures were higher in group 2 on the first and second postoperative days, and there was no difference in the preoperative assessment (Table 2).

**Table 2:** Comparison of maximal respiratory pressure measurements in the preoperative, 1 ^st^ postoperative, and 2 ^nd^ postoperative evaluations.

MRP	Group 1 (n=27)	Group 2 (n=28)	p
MIP (Pre-op)	-74±20	-86±21	0.03
MIP (1^st^ PO)	-50±21	-78±28	<0.0001
MIP (2^nd^ PO)	-59±18	-81±23	<0.0001
MEP (Pre-op)	81±19	85±18	0.27
MEP (1^st^ PO)	51±15	75±22	<0.0001
MEP (2^nd^ PO)	65±14	81±19	<0.0001

MRP: maximum respiratory pressures; MIP: maximum inspiratory pressure; Pre-op: preoperative; PO: postoperative; MEP: maximum expiratory pressure.

In the intragroup evaluation, the MIP measurements of group 1 were higher in the preoperative period when compared with the first postoperative day. We observed the same when comparing the first with the second postoperative days. In group 2, there was no difference in MIP when comparing the preoperative period and the first and second postoperative days (Table 3). The behavior of MEP measurements was similar. In Group 1, they were higher preoperatively when compared with the first postoperative day and were also higher when compared with the first and second postoperative days (Table 4). In group 2, there was no difference in MIP measurements when comparing the preoperative period with the first and second postoperative days (Table 4).

**Table 3:** Comparison of MIP measurements in the preoperative, 1 ^st^ postoperative, and 2 ^nd^ postoperative evaluations.

MIP	Group 1 (n=27)	p	Group 2 (n=28)	p
Pre-op	-74±20	<0.0001	-86±21	0.30
1^st^ PO	-50±21		-78±28	
Pre-op	-74±20	0.005	-86±21	0.37
2^nd^ PO	-59±18		-81±23	
1^st^ PO	-50±21	0.02	-78±28	0.64
2^nd^ PO	-59±18		-81±23	

MIP: maximum inspiratory pressure; Pre-op: preoperative; PO: postoperative.

**Table 4:** Comparison of MEP measurements in the preoperative, 1 ^st^ postoperative and 2 ^nd^ postoperative days.

MEP	Group 1 (n=27)	p	Group 2 (n=28)	p
Pre-op	81±19	<0.0001	85±18	0.27
1^st^ PO	51±15		75±22	
Pre-op	81±19	0.004	85±18	0.60
2^nd^ PO	65±14		81±19	
1^st^ PO	51±15	0.003	75±22	0.18
2^nd^ PO	65±14		81±19	

MEP: maximum expiratory pressure; Pre-op: preoperative; PO: postoperative.

As for the visual analogue pain scale (VAS), group 1 had more severe pain than group 2, both on the first and second postoperative days (Table 5). In the intragroup analysis, there was no difference between the first and second postoperative days in group 1. In group 2, pain was more intense on the first postoperative day (Table 6).

**Table 5:** Comparison of the visual analogue pain scale at the first and second postoperative days..

	VAS		
	Group 1 (n=27)	Group 2 (n=28)	p
1^st^ PO	2.48±1.34	1.36±1.37	0.003
2^nd^ PO	1.33±1.11	0.61±0.79	0.01

VAS: visual analogue pain scale; PO=postoperative.

**Table 6:** Comparison of the visual analogue pain scale at the first and second postoperative days.

	EVE		
	1^st^ PO	2^nd^ PO	p
Group 1 (n=27)	2.48±1.34	1.33±1.11	0.001
Group 2 (n=28)	1.36±1.37	0.61±0.79	0.05

VAS: visual analogue pain scale; PO=postoperative.

## DISCUSSION

Respiratory muscle strength in obese individuals is a frequent object of analysis and research, but studies to calculate MIP and MEP reference values in obese individuals are divergent. Pouwels et al.^16^ evaluated the respiratory muscle strength of 122 morbidly obese patients before and after bariatric surgery and compared these estimates with predictive values calculated using five different mathematical equations. In the preoperative period, they found only one non-divergent result of the MIP measured in relation to the calculated pressure. In the postoperative evaluation, all the measured MIP values were different from the calculated values. Pazzianotto-Forti et al.^12^, in a study like that of Pouwels, observed that the values obtained and calculated for MEP were not in agreement in morbidly obese patients. In the present study, although there were no demographic and anthropometric differences between the studied groups, we observed that in the preoperative period the MIP of the laparoscopic group was significantly different when compared with the conventional approach group. Azevedo et al.^17^, in a review of reference values for respiratory muscle strength in Brazilians, showed that, in addition to the biological characteristics of populations contributing to the expressive variability in the values of maximum respiratory pressures between individuals, individual factors such as physical fitness and the degree of schooling can also impact results.

An important point to be considered in patients undergoing abdominal surgery via laparotomy is the impact of the surgical incision. Ventilatory mechanics are altered, and pain is also a limiting factor for lung reexpansion. These factors are associated with pulmonary complications in up to 30% of cases^8^. In our study, the laparotomy group had a significant reduction in respiratory muscle strength after the procedure. On the second postoperative day, the day of hospital discharge, it still had not returned to preoperative values. Paisini et al.^18^ evaluated respiratory muscle strength in a sample similar to ours and found reduced inspiratory and expiratory pressures up to the fifth postoperative day. Parreira et al.^19^ evaluated respiratory muscle strength 36 months after bariatric surgery. They demonstrated a significant increase in inspiratory muscle strength and a return to preoperative values of expiratory muscle strength. Cavalcanti et al.^20^ also demonstrated a reduction in MIP in the postoperative period of gastroplasty and analyzed the repercussion of conventional respiratory physiotherapy and non-invasive ventilation with two airway pressure levels. They pointed out that on the third postoperative day, MIP had not returned to baseline values in both studied groups. Casali et al.^21^ and Rocha et al.^22^ demonstrated that exercises with inspiratory load favor the return of MIP to baseline values in the postoperative period of bariatric surgery.

An important finding of the present study was the behavior of maximal respiratory pressures in the group undergoing laparoscopy. We found no difference between the values of MIP and MEP in the preoperative period in relation to the values found in the postoperative period. Huisstede et al.^10^ published a study involving 485 patients undergoing laparoscopic gastroplasty. Patients who developed postoperative pulmonary complications had significantly lower preoperative spirometric values when compared with individuals without complications. Remístico et al.^23^ also demonstrated a reduction in spirometric variables in a clinical trial with 30 patients undergoing laparoscopic reduction gastroplasty. However, in both studies, respiratory muscle strength was not evaluated. Barbalho and Moulim et al.^24^, when evaluating maximal respiratory pressures in a study comparing bariatric surgery performed by laparotomy and laparoscopy, showed that respiratory muscle strength was affected in both groups, being more pronounced in the laparotomy group. Cohen et al.^25^, in a review article on the systemic changes caused by laparoscopy, described that the best-documented physiological benefit is the preservation of pulmonary function in the postoperative period. In our study, there was no incidence of pulmonary complications during the study period in both groups. This may be one of the factors that explain the lack of difference in manovacuometry measurements in the preoperative period in relation to the postoperative period in the group operated by the laparoscopic approach. Other factors that can be considered are the reduced surgery time, 57 minutes on average, postoperative physical therapy, and the stimulus for early hospital discharge, which occurred two days after surgery. These findings were similar to those found in the study by Barbalho and Moulim^24^. In addition, postoperative pain intensity plays an important role in preserving pulmonary function^26^ ^-^ ^28^. Shobary et al.^29^ compared the intensity of pain in gastric bypass by the laparoscopic approach in relation to the conventional approach. They showed that patients operated by the laparoscopic route had lower pain scores at rest and in movement and shorter surgical time, corroborating the findings of the present study.

## CONCLUSION

In bariatric surgery using the conventional access route, there is a reduction in maximal respiratory pressures during the surgical stay, with no return to baseline values two after the procedure (discharge day). In the laparoscopic route, there is no change in respiratory muscle strength when comparing preoperative and postoperative values. In addition, laparoscopy leads to less severe pain during surgical hospitalization.
